# Therapeutical and Pharmaceutical Notes

**Published:** 1901-09

**Authors:** W. J. Martin

**Affiliations:** Kankakee, Ill.


### THERAPEUTICAL AND PHARMACEUTICAL NOTES.

UNDER THE DIRECTION OF W. J. MARTIN, M.D.C.,
KANKAKEE, ILL.

---

A NEW ANTIDOTE FOR MORPHINE POISONING. E. T. Reichert, M.D., professor of physiology in the University of Pennsylvania, has been experimenting with adrenalin (the newly discovered active principle of the suprarenal gland) for the purpose of observing its action upon the internal secretion of both healthy and morphinized dogs. Observing, first, that morphine, even in small doses, profoundly depresses general metabolism, he reasoned that the lethal action of morphine could be overcome by the administration of a circulatory, respiratory, and general metabolic stimulant. Such a stimulant was adrenalin. As the result of his experiments he declares (*University of Pennsylvania Medical Bulletin*) that "the positive and prompt action of adrenalin upon the respiratory movements, heart, arterial pressure, general metabolism, and body temperature justify the belief that this substance will be found of value in opium and morphine poisoning, in failure of the circulation, in the prevention of collapse in anæsthesia, and in allied conditions. It is probable, owing to its powerful local action as a vasoconstrictor, that abscesses will be caused by subcutaneous injections. If given by the stomach in morphinized individuals it should be administered with alcohol in some form so as to increase the rapidity of absorption." Reduced to its simplest terms, adrenalin prevents or overcomes the fall of temperature and the decrease in metabolism which morphine causes, and is thus a direct physiological antidote.—*Bulletin Phar.*

STERILIZING SPONGES. As is well known, it is a rather difficult matter to completely sterilize sponges; in fact, when boiled, whether in pure water or in alkaline or carbolized water, the sponges lose their elasticity and absorbent power. Elsberg (*L'Union Phar.*) has, however, found a method of perfectly sterilizing them without in any way impairing their properties. The sponges are first immersed for two days in diluted hydrochloric acid to remove all calcareous matter, then carefully washed with cold water, and boiled for fifteen minutes in a solution of the following composition: Potassa, 1 part; tannic acid, 3 parts; water, 100 parts. It only remains to rinse the sponges in a suitable antiseptic solution and preserve them in a 5 per cent. carbolic acid solution.—*Ibid.*

PHENOL IN ACUTE ARTICULAR RHEUMATISM. A. Bolduzzi injects 1 c.c. of a 3 per cent. solution of phenol into the most tumefied joint or joints. A cure has promptly resulted in the cases so treated, confirming the assumption of the etiological relationship between this disease and erysipelas, in which latter the subcutaneous injection of phenol is equally effective. The above treatment is well worthy of trial in the treatment of articular arthritis so common in adult horses and young foals.—W. J. M.

RETENTION OF STITCHES IN SUPPURATING WOUNDS. Where wounds become infected after suture Weir (*Med. Summary*) leaves in a few stitches for the purpose of retention as long as suppuration does not necessitate their removal. He believes their value in retaining the edges of the wound offsets the harm they do in keeping up the inflammation.

PROGRESS OF VETERINARY LITERATURE. *Clinical Veterinary Medicine and Surgery.* By Prof. P. J. Cadiot. Translated by John A. W. Dollar, M.R.C.V.S. This is a work of over 600 pages, and is divided into parts or sections, the majority of which consist of lectures and clinical demonstrations given by Prof. Cadiot to the veterinary students of the veterinary school at Alfort. The work, as a whole, is an excellent example of the exact and scientific manner in which that able and painstaking veterinarian performs all his work. The balance of the work consists of numerous reports of cases met with by Dollar in his practice or taken by him from the files of English veterinary periodicals. In the past there has been a woful lack of reference works in the English language on the subject of clinical veterinary medicine and surgery, and the appearance of such works has always been hailed by English speaking veterinarians as a distinct addition to the all too meagre literature of our otherwise rapidly advancing science. The work under consideration is well illustrated with numerous woodcuts, which serve to make clear to the reader the *modus operandi* of performing the various operations described in the text. The work describes in a clear and concise manner the methods of performing nearly every known surgical operation performed upon domestic animals, and also gives a very full account of the proper therapeutic measures to be carried out after such operations. Among the more important operations described is that of cryptorchidotomy as peformed on the horse by the Belgian method; neurectomy of the median and ulnar nerves; surgical treatment of chronic roaring; Bosi's operation of neurectomy for spavin; amputation of the penis, etc. A complete account is given of the history, etiology, and treatment of contagious equine pneumonia; also of hæmoglobinuria (azoturia) of the horse, together with exophthalmic goitre of the same animal; eczema in the dog and horse, and tuberculosis in the canine, feline, equine, and avian species.

In the chapter on tuberculosis of the cat the author gives an interesting account of the various manners in which that animal contracts the disease. The author states that, contrary to the general opinion, three-fourths of the causes of tuberculosis among cats are due to infection received from persons suffering from tuberculosis. This statement coincides with my own observations extending over a period of several years, during which I have observed that in families in which tuberculosis is prevalent you will almost invariably find tuberculosis to prevail among the feline members of such a family. There has been heretofore a generally accepted opinion among veterinarians that tuberculosis is a rare disease among horses. Prof. Cadiot gives a very full account of this disease as it exists in the horse. He says: "Bearing in mind the varying forms which tuberculosis may assume in the horse, it is rare that some of the complex assemblage of symptoms fails to suggest the correct diagnosis. The final conclusion is assisted by auscultation, percussion, rectal exploration, and palpation of accessible lymphatic glands, and is confirmed by bacteriological examination, and the injection of tuberculin or inoculation."

Prof. Cadiot states that the injection of 30 cgs. of tuberculin is followed in a tuberculous horse by a reaction, which usually attains its maximum about the fifteenth hour, the temperature rising about 2° or 3° C. M. Cadiot also states that the proportion of pulmonary tuberculosis in the horse is about 70 per cent.; tuberculosis of the mesenteric and sublumbar glands and the spleen, about 40 per cent.; of the liver, pleura, and peritoneum, 20 per cent.; of the intestines, 15 per cent. He also states that tuberculosis of the kidneys is rare in horses.

Part V. of the work contains an interesting account of the treatment of tuberculosis in the guinea-pig with the parotid saliva of horses collected aseptically. Although these experimental injections of parotid saliva failed to produce any beneficial therapeutic effects it nevertheless serves to show with what tireless energy scientific investigators are seeking an antidote that will stay the deadly ravages of the great white plague—tuberculosis. The work also contains an account of the serum treatment of glanders carried out by the author and other European investigators, also an account of his experiments with vanadine used subcutaneously as a therapeutic agent in the treatment of pneumonia in the horse and dog, in the abdominal form of influenza, distemper, and other forms of wasting diseases, also an account of the author's experience with iodine used intravenously as a therapeutic agent in various diseases.

This work, containing as it does the ripe surgical and therapeutical experience of one of the foremost veterinary clinicians and teachers of the day, contains a vast amount of exact scientific information of the utmost benefit to the busy workaday practitioner, while for the student of either human or comparative medicine no better book could be placed in their hands that will give them a clear insight into the many intricate problems with which they are daily confronted.

The paper, printing, and binding of the work is all the most critical could desire, and is a credit to the firm of W. R. Jenkins.

Text-book of Veterinary Medicine. By James Law, F.R.C.V.S. This work is designed by the author to be a text-book on the principles and practice of veterinary medicine. The intention is to complete the work in four volumes, which will include a complete description of all the diseases of domestic animals usually met with in veterinary practice. Vol. I., which was issued in 1896, includes the study of general and special pathology, pathological chemistry, etiology, symptomatology, medical diagnosis, and prognosis of diseases. In it are also described diseases of the respiratory organs, diseases of the heart and the organs of circulation, acute and infectious inflammatory diseases of the lymphatic system, etc. Vol. II., issued in 1900, gives a full description of the diseases of the digestive organs—liver, spleen, pancreas, etc. Vol. III., which is but recently off the press, includes the diseases of the nervous system, genito-urinary organs, the eye, skin, and the constitutional diseases. Vol. IV., which will soon be issued, will include a description of the infectious diseases, sanitary science, parasites, and parasitisms.

Not since Prof. W. Williams issued his monumental work on the principles and practice of veterinary medicine has there been placed before the members of the veterinary profession so complete and scientific an exposition of the diseases of domestic animals as can be found within the covers of Prof. Law's work. It bears on every page evidence of deep study, careful and laborious research, a grouping and presentation of the latest scientific advancement in knowledge, together with the most approved modern methods employed in the treatment of animal diseases. Published by the author, Ithaca, New York.

Effects of Salicylates upon Metabolism. F. W. Goodbody (*Journ. Physiol.*) finds, as the result of recent investigations, that sodium salicylate causes an increase in the quantity of urine excreted and in the specific gravity, the latter increase being principally due to the increased elimination of the nitrogenous substances, especially of the urea. The drug does not seem to have any influence on general metabolism so far as the absorption of proteids and fats is concerned, but it increases the breaking up of proteids in the body.

Elimination of Antipyrine. According to D. Lawrow (*Zeit. Phys. Chem.*) antipyrine is eliminated by the body in its original form to only a small percentage. Rather is the same given off in the form of a compound of glucoronic acid, which is supposed to result from a combination of glucoronic acid and an oxyantipyrine.

For uræmia supervening after an attack of acute nephritis the subcutaneous injection of normal salt solution will be found of decided benefit.

Thomas Sydenham, originator of Sydenham's laudanum or wine of opium, and one of the founders of the science of pharmacology, was born in 1624 and died in 1689.

Iodide of Potassium Treatment of Human Actinomycosis. Lieblein (*Beiträge zur klinischen Chirurgie*, Band xxviii., Heft 1) offers a much needed critique of the status of iodide of potassium as a curative agent in human actinomycosis Sixty-two reported cases are analyzed, of which forty-two were healed, seven improved, successfully treated one without success, incomplete four, deaths six, the jaw being most frequently represented, and the lung and intestine in about equal number. The iodide of potassium is most efficient in jaw actinomycosis, least in the pulmonary form. Where iodide of potassium alone was relied upon three deaths ensued; but these were too advanced to be even benefited by operative treatment.

Of greater interest are observations on the use of iodide of potassium after unsuccessful operative interference. Once death supervened, once marked improvement, and three times cure. One of the last group was twice ineffectually operated on. The necessity of operative interference after unsuccessful application of iodide treatment arose three times. In these latter instances the iodide was given for too short a time.

At this point the author passes on to the method of giving iodide of potassium. It was only found serviceable when given for several months or more. Treatment for one year or more was only called for in severest cases.

As to the modes of action of iodide of potassium, it is pointed out that by the disintegration of the cellular infiltration and its discharge through fistulous tracts the expulsion of the ray fungus is effected. In support of this theory is the bacteriological observation that iodide of potassium does not inhibit the growth of the ray fungus, and coupled to this is the clinical observation that the cases with sinuses coexisting come to a more speedy termination under iodide of potassium treatment. The advent of any mixed infection retards and even wholly prevents beneficent action of iodide of potassium. The quantity of iodide of potassium given fluctuated between 100 and 300 grammes, one case taking *in toto* 4000 grammes. Recurrences were only apparent because of insufficient treatment, and disappeared wholly when properly administered. But recurrence also took place after operation, no matter how seemingly thorough it was done.

Limitations of iodide of potassium treatment, because of its slowness of action, exist when the disease is of an acute phlegmonous character and when the individual is very cachectic. Here it is merely an adjuvant to surgical measures. In these respects we have analogous conditions in syphilis. Thus, while iodide of potassium is no specific, it is a remedial of no mean order antagonistic to actinomycosis, and worthy of an extended trial in proper doses for a long period in every instance. Thus, in Wolfler's clinic it is the practice to give three grammes in increasing doses three times daily. The affected area is also covered with compresses of iodide of potassium or a tract or cavity tamponed with iodide of potassium gauze. The diminution in size of swelling is ever slow but steady.—*Annals of Surgery*, March, 1901.

Dr. Ritchie contributes some important observations on the bacteriology of bronchitis to the current number of the *Journal of Pathology.* The main points may be thus summarized :

1. Acute bronchitis is an infective disease.

2. It is not due to any specific microörganism ; various bacteria are found in the bronchitic secretion.

3. Some of these bacteria are exciting causes of the bronchitis.

4. The disease is more often due to mixed infection than to the action of one form of bacterium alone.

5. The most important causal bacteria are diplococcus pneumoniæ and streptococci.

6. The influenza bacillus not infrequently causes bronchitis, apart from epidemic influenza.

The thermometer was invented by Galileo about the year 1595, and 1611 Sanctonius first used Galileo's thermometer as an aid in determining the temperature of fever patients.

